# B‐Mode Ultrasonographic Evaluation and Reference Dimensions of the Liver and Spleen in Healthy Lactating Saanen Goats

**DOI:** 10.1111/vru.70159

**Published:** 2026-03-29

**Authors:** Tatiane Vitor da Silva, Isabela Bernardes Moreira, Raissa da Silva Carvalho, Clara Beatriz de Avila Santiago, Mário Felipe Alvarez Balaro

**Affiliations:** ^1^ Faculdade de Veterinária Universidade Federal Fluminense Niterói Rio de Janeiro Brazil; ^2^ Universidade Federal de Minas Gerais Belo Horizonte Minas Gerais Brazil

**Keywords:** B‐mode ultrasonography, diagnostic imaging, reference values, small ruminants

## Abstract

This study aimed to establish reference ultrasonographic parameters for the liver and spleen in 34 healthy Saanen goats. The spleen was visualized between the 12th and 8th intercostal spaces (ICS), with homogeneous parenchyma and a hyperechoic capsule and splenic vein with a mean diameter of 3.3 ± 0.9 mm. The liver was observed between the 12th and 6th ICS, showing a hypoechoic, homogeneous parenchyma. The portal vein and caudal vena had average diameters of 16.5 ± 2.6 and 14.1 ± 3.4 mm, respectively. The gallbladder exhibited variable shapes with anechoic content in 73.5% (25/34) of the animals.

## 1 Introduction

Abdominal ultrasonography has become an essential diagnostic modality in veterinary medicine, allowing noninvasive, real‐time assessment of internal organs in both large and small animal practices. The first studies on liver and spleen ultrasonography in ruminants were conducted in cattle during the 1990s [1, 2], and since then, similar investigations have been extended to other species, including calves [3], buffaloes [4], camels [5], and small ruminants [6].

The incorporation of ultrasonography into routine clinical practice has enabled veterinarians to evaluate organ size, shape, echotexture, margins, and internal architecture in a continuous, noninvasive, rapid, and safe manner for both animals and operators [7, 8].

This is particularly relevant in clinical settings, where hepatic biochemical changes often appear only after more than 50% of the liver parenchyma is compromised, and no specific clinical signs or laboratory markers exist to reliably indicate splenic abnormalities [9].

Despite this potential, there is a paucity of systematic studies describing the normal ultrasonographic appearance and biometric parameters of the spleen and liver in goats under controlled conditions. Therefore, this study aimed to describe the ultrasonographic and biometric characteristics of the liver and spleen in clinically healthy lactating Saanen goats. This work seeks to support more accurate diagnoses and improve early detection of hepatosplenic disorders in caprine medicine.

## 2 Materials and Methods

### 2.1 Ethics

All procedures, described in this study, were approved by the Ethics Committee for Animal Use of the Fluminense Federal University (Protocol No. 2919170821) and conducted in accordance with the ethical guidelines of the *Sociedade Brasileira de Experimentação Animal* and the ARRIVE (Animal Research: Reporting of In Vivo Experiments) guidelines [10].

### 2.2 Study Location and Experimental Conditions

The study was carried out on a commercial dairy goat farm located in Sapucaia, Rio de Janeiro, Brazil. The entire flock was routinely vaccinated against clostridiosis and rabies and dewormed according to a standardized flock health protocol. The goats were raised under an intensive management system and fed four times daily with a total mixed ration (TMR) containing 82% dry matter, composed of capiaçu silage (67.9%) and barley (14.2%). The remaining 17.9% consisted of concentrate made from cornmeal (66.5%), soybean meal (28%), Bovigold mineral supplement (2.5%), urea (1%), and white salt (1%). Fresh water and a goat‐specific mineral salt supplement were provided ad libitum.

### 2.3 Animals and Health Status

A total of 34 lactating Saanen goats were included in the study (3.75 ± 1.0 years of age; body condition score [BCS] 2.6 ± 0.8 on a 1–5 scale; [11]), with a mean live weight of 53.3 ± 9.4 kg, 254 ± 89.5 days in lactation, and an average daily milk production of 2.5 ± 0.8 L. To confirm the health status of the animals, all underwent a comprehensive clinical examination as described by Pugh et al. [12], along with laboratory testing that included complete blood count and biochemical analyses of renal and hepatic function.

### 2.4 B‐Mode Ultrasonography

B‐mode ultrasonographic evaluations of the spleen and liver were performed by a single operator, with each animal examined once. A portable B‐mode ultrasound unit (SonoScape S6, SonoScape, Shenzhen, China) equipped with a 5.0 MHz convex transducer was used. Device settings were standardized and maintained throughout the study (frames per second: 28; depth: 8.9 cm; gain: 146; power: 80%; focal zones: 2–3 cm apart).

Initially, hair was removed from the thoracic region and from the right and left flanks, and conductive gel was applied to improve contact between the transducer and the skin. All animals were examined while standing, without sedation, to minimize stress, preserve physiological organ positioning, and ensure animal welfare.

**FIGURE 1:**
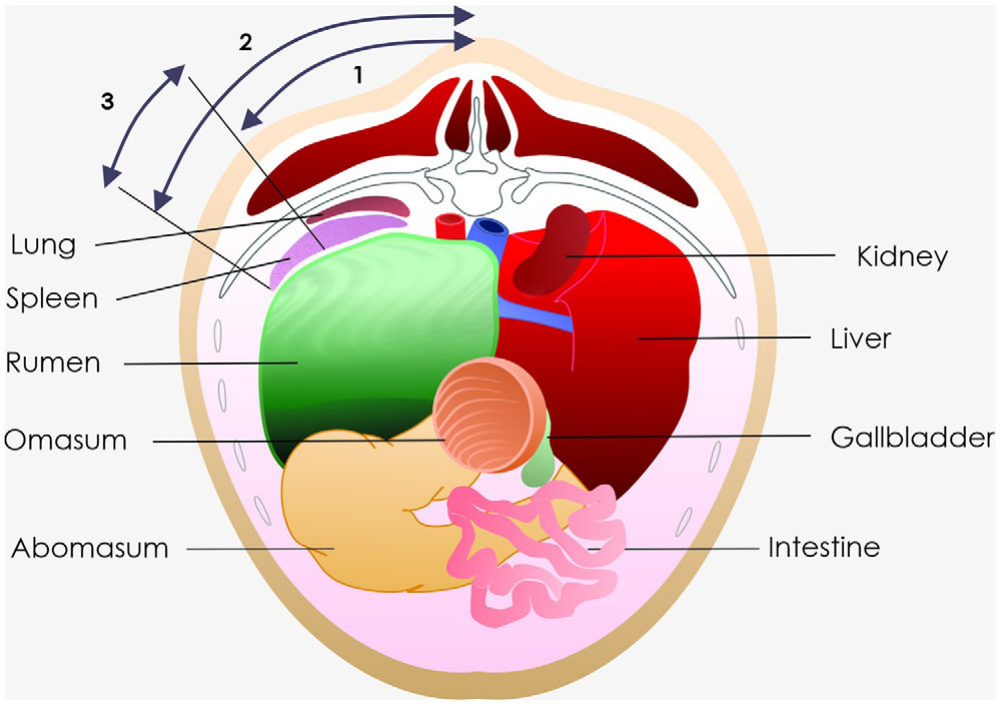
Schematic representation of spleen size determination in cross‐section: (1) distance between the midline of the back and the dorsal surface of the spleen; (2) distance between the midline of the back and the ventral surface of the spleen; and (3) size of the spleen. Left abdomen is on the left side of the image. *Source*: Adapted from [16].

The spleen ultrasound was initiated caudal to the last rib and extended cranially to the eighth intercostal space (ICS) on the left side. The transducer was positioned parallel to the ribs and moved in dorsal‐to‐ventral and caudo‐cranial directions, following the protocol described by Floeck et al. [13] and Da Silva et al. [14]. The dorsal margin distance (DMD) and ventral margin distance (VMD) were determined by measuring from the spinous processes to each respective splenic border, using the dorsal midline as a reference point in each ICS (Figure 1). Once the spleen margins were visualized and centered on the ultrasound image, the transducer's central position was marked on the skin, and distances from the dorsal and ventral splenic edges to the midline were measured using a measuring tape. The visible splenic thickness in each ICS was calculated by subtracting the DMD from the VMD, as described by Floeck et al. [13]. Splenic length was also measured in each ICS using the ultrasound device's caliper function on a frozen image [15].

**FIGURE 2:**
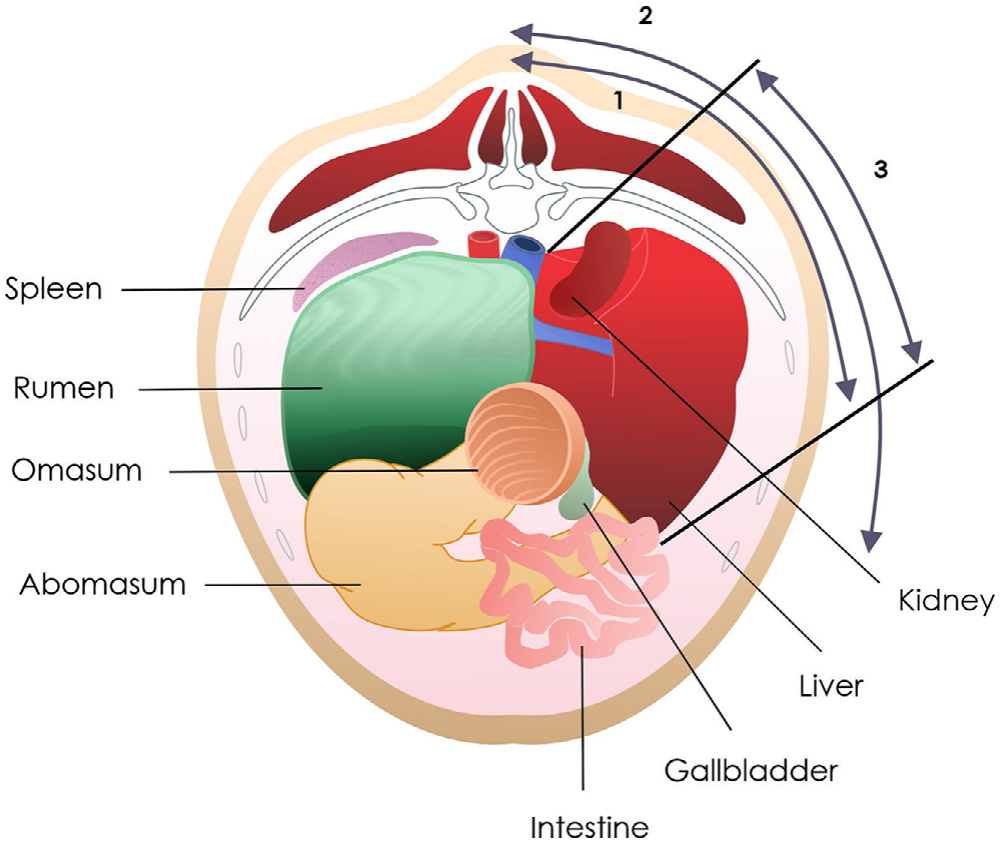
Schematic representation of the liver size determination in cross‐section: (1) distance between the dorsal margin of the liver and the dorsal midline; (2) distance between the ventral margin of the liver and the dorsal midline; and (3) visible extension of the liver. Left abdomen is on the left side of the image. *Source*: Adapted from [17].

The liver ultrasound was performed on the right side, starting caudal to the last rib and progressing cranially to the sixth ICS. The transducer was held parallel to the ribs and moved in dorsal‐to‐ventral and caudo‐cranial directions. The hepatic parenchyma was evaluated, as well as the gallbladder when visible, regarding its location, shape, and contents. The area and diameters of the portal vein (PV) and caudal vena cava (CVC) were measured at the 11th ICS. Hepatic extent and length were assessed using a methodology similar to that applied for the spleen (Figure 2). A comparative assessment of hepatic and splenic echogenicity was also performed.

### 2.5 Statistical Analysis

Data were organized in electronic spreadsheets and analyzed using descriptive statistics. Quantitative variables with a normal distribution were expressed as arithmetic means ± standard deviations (SD), whereas ordinal data were presented as medians with interquartile ranges (IQR). Categorical variables were expressed as absolute frequencies or percentages, as appropriate.

## 3 Results

### 3.1 Hematological and Biochemical Results

All hematological and biochemical parameters remained within the reference ranges established for goats [18]. The mean values were as follows: urea, 42.1 ± 10.8 mg/dL; creatinine, 0.6 ± 0.1 mg/dL; aspartate aminotransferase (AST), 102.4 ± 20.1 IU/L; alkaline phosphatase (ALP), 249.3 ± 64.0 IU/L; total protein, 7.1 ± 0.7 g/dL; albumin, 3.0 ± 0.2 g/dL; globulin, 4.0 ± 1.0 g/dL; calcium, 8.6 ± 1.3 mg/dL; and phosphorus, 7.8 ± 2.6 mg/dL.

### 3.2 Spleen

**FIGURE 3:**
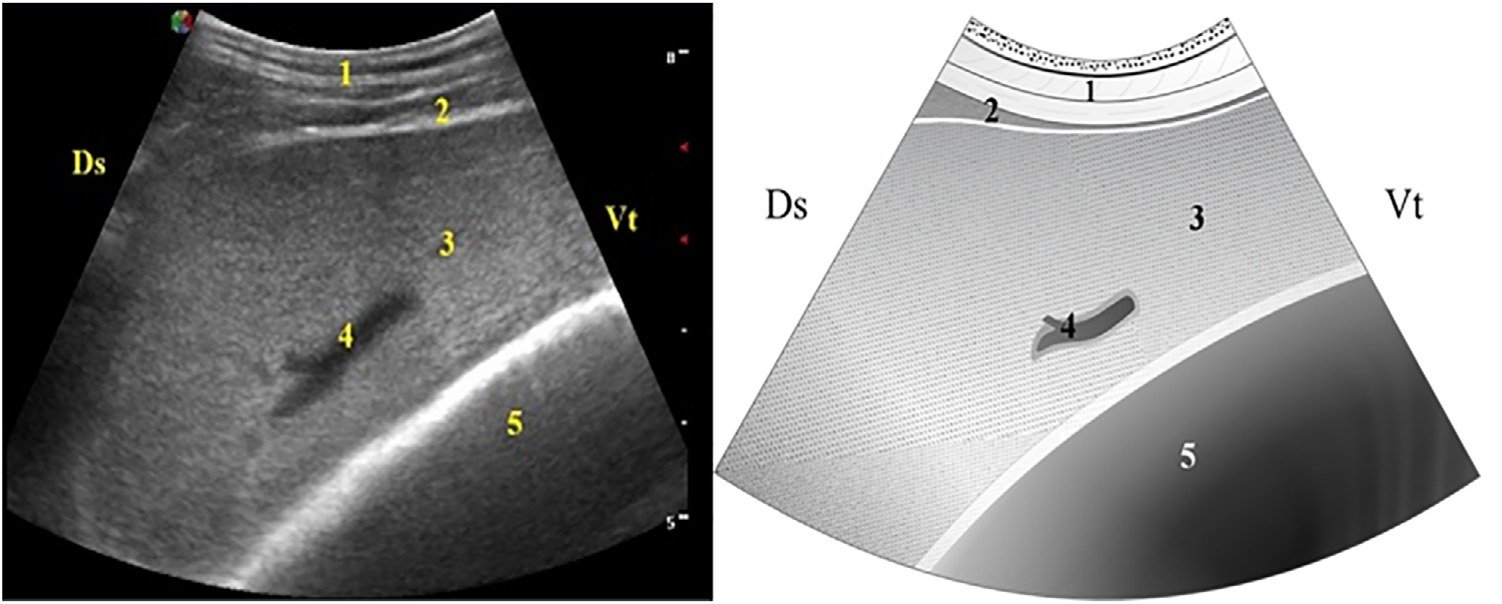
Ultrasonogram and schematic representation of the splenic parenchyma: (1) lateral abdominal wall; (2) splenic capsule; (3) splenic parenchyma; (4) splenic vein; and (5) rumen. Ds, dorsal; Vt, ventral.

The splenic parenchyma was visualized from the caudal aspect of the last rib to the eighth ICS on the left side. It appeared as a finely granular structure with a homogeneous echotexture surrounded by a thin, smooth, hyperechoic capsule with a mean thickness of 0.85 ± 0.24 mm and a clearly visible splenic vein inserted within it (Figure 3). In all animals, the spleen was visualized between the 12th and 9th ICS, whereas it extended to the eighth ICS in only 23.5% (8/34) of the goats.

The splenic vein was identified as an anechoic structure ranging from elongated to circular in shape. It was observed between the 12th and 9th ICS, with more frequent visualization between the 11th and 10th ICS. The mean vein diameter was 3.3 ± 0.9 mm.

The distance from the dorsal margin of the spleen to the dorsal midline was smallest caudal to the last rib and gradually increased cranially, reaching the highest values at the ninth and eighth ICS. A similar pattern was observed for the ventral margin, with the largest values also found at the ninth and eighth ICS and the smallest values caudal to the last rib. The maximum splenic thickness was recorded at the 11th ICS (Table 1).

**TABLE 1:** Location of the spleen vein, mean ± SD, spleen measurements in 34 healthy Saanen goats using a 5.0 MHz convex transducer.

Variable	Caudal to last rib	12th ICS	11th ICS	10th ICS	9th ICS	8th ICS
No of goats	18	34	34	34	31	8
Localization of the splenic vein	—	13	34	25	3	1
DMD (cm)	10.11 ± 2.47	11.57 ± 2.20	13.51 ± 2.50	15.49 ± 2.67	18.22 ± 3.0	22.22 ± 2.64
DMV (cm)	14.90 ± 2.08	16.14 ± 2.03	18.91 ± 2.08	20.86 ± 2.51	23.47 ± 3.04	26.78 ± 2.11
Size (cm)	4.79 ± 1.58	4.60 ± 1.70	5.54 ± 2.08	5.40 ± 2.0	5.0 ± 2.36	4.56 ± 1.51
Thickness (cm)	2.46 ± 0.8	3.35 ± 0.9	4.52 ± 0.86	4.26 ± 0.9	2.97 ± 1.02	2.90 ± 1.03

Abbreviations: DMD, dorsal margin distance; ICS, intercostal space; SD, standard deviations.

### 3.3 Liver

**FIGURE 4:**
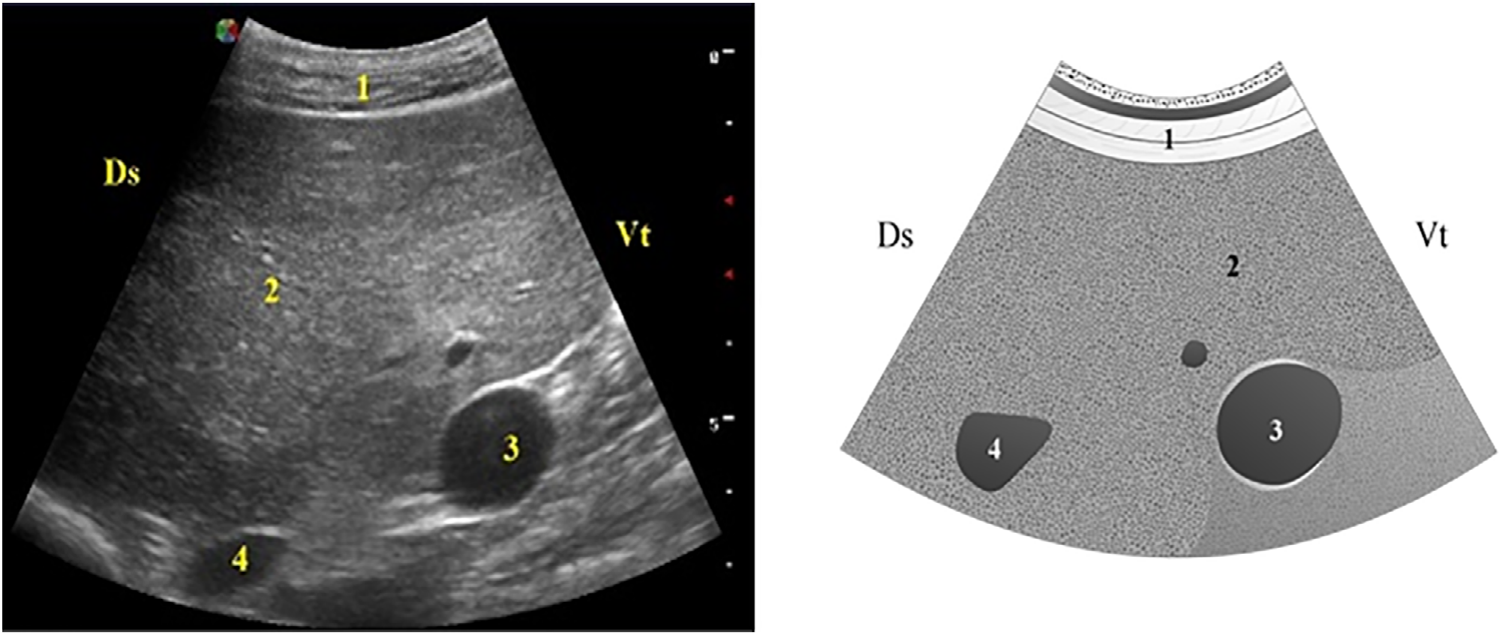
Ultrasonographic image of the liver: (1) lateral abdominal wall; (2) liver parenchyma; (3) portal vein; and (4) caudal vena cava. Ds, dorsal; Vt, ventral.

The liver was visualized in all animals, presenting a homogeneous hypoechoic parenchyma with blood vessels embedded within (Figure 4). Anechoic tubular structures delineated by hyperechoic walls corresponding to bile ducts were also observed. The liver was consistently seen from the 12th to the 7th ICS and extended to the 6th ICS in 73.5% (25/34) of the goats.

In a comparative analysis of echogenicity, the liver appeared hypoechoic relative to the spleen in 76.5% (26/34) of the animals and isoechoic in 23.5% (8/34).

The DMD from the liver to the midline was greater at the eighth, seventh, and sixth ICS, whereas the VMD was smallest at the 12th ICS and increased progressively in the cranial direction. The largest longitudinal extension of the liver was noted at the 10th and 11th ICS (Table 2).

**TABLE 2:** Mean ± SD sonographic liver measurements in 34 healthy Saanen goats using a 5.0 MHz convex transducer.

Variable	12th ICS	11th ICS	10th ICS	9th ICS	8th ICS	7th ICS	6th ICS
No of goats	34	34	34	34	34	34	26
DMD (cm)	12.0 ± 1.44	13.17 ± 2.38	14.91 ± 2.73	10.0 ± 2.83	22.94 ± 3.0	27.51 ± 3.29	31.33 ± 3.01
DMV (cm)	18.91 ± 2.54	21.26 ± 2.34	25.46 ± 2.83	28.34 ± 2.54	30.94 ± 2.68	33.23 ± 2.74	35.85 ± 2.94
Size (cm)	6.81 ± 3.23	8.09 ± 3.11	10.49 ± 2.72	10.74 ± 3.32	8.0 ± 2.96	5.80 ± 2.31	4.59 ± 1.74
Thickness (cm)	5.54 ± 0.90	6.04 ± 0.95	6.15 ± 0.90	5.10 ± 0.73	5.02 ± 7.77	4.55 ± 1.06	2.96 ± 1.10

Abbreviations: DMD, dorsal margin distance; ICS, intercostal space; SD, standard deviations.

The PV was visualized as a circular anechoic structure with an echogenic wall and a characteristic stellate branching pattern within the hepatic parenchyma. Its mean area and diameter were 209.3 ± 70.5 mm^2^ and 16.5 ± 2.6 mm, respectively. The CVC exhibited a triangular shape in 91.2% (31/34) and a rounded shape in 8.8% (3/34) of the animals, with an anechoic lumen. Its mean diameter was 14.1 ± 3.4 mm, and its mean area was 126.8 ± 61.4 mm^2^.

Both the PV and CVC were consistently visualized between the 12th and 8th ICS, with the optimal imaging windows found at the 11th and 10th ICS, where both vessels were observed in all animals.

The gallbladder exhibited variable shapes, circular, oval, and pear‐shaped, and was observed between the 10th and 8th ICS. The best acoustic window was noted at the 10th ICS in 52.9% (18/34) of the goats. The gallbladder presented anechoic content in 73.5% (25/34) of the animals and hypoechoic content in 8.8% (3/34); it was not visualized in 17.6% (6/34). Gallbladder length ranged from 24.2 to 78.2 mm (mean: 44.8 ± 12.3 mm), and height ranged from 6.1 to 41.9 mm (mean: 17.1 ± 8.8 mm). The gallbladder wall has an average thickness of 1.3 ± 0.4 mm.

## 4 Discussion

The topographical localization of the spleen in healthy goats has been previously described by Braun and Steininger [16] and Balasundaram and Sivagnanam [15], with findings similar to those of the present study. Ultrasonographic mapping of splenic position is clinically valuable, as traditional semiological techniques, such as percussion and palpation, are imprecise in small ruminants due to the dorsal location and thin profile of the organ, which reduces acoustic and tactile feedback.

In healthy goats, the spleen appears as a hyperechoic structure with fine, homogeneous granular echotexture, surrounded by a thin, smooth, and hyperechoic capsule [19]. Recognizing this typical appearance is clinically important, as disease processes can alter normal splenic echotexture and echogenicity.

Recognizing this typical appearance is clinically important, as inflammatory processes may alter splenic echogenicity, producing heterogeneous patterns or hyperechoic foci.

The splenic vein in this study was observed as an anechoic, circular, or elongated structure between the 12th and 9th ICS, consistent with previous descriptions in calves [20], sheep [14], and goats [15]. This pattern is typical of healthy animals. However, alterations such as intraluminal echogenic material may indicate splenic vein thrombosis [21], and venous dilation can suggest early vascular compromise. These findings highlight the diagnostic potential of routine splenic vein assessment, especially in suspected cases of thromboembolic disease or venous congestion [22, 23].

The liver was consistently visualized between the 12th and 7th ICS and extended to the 6th ICS in over 70% of the goats. This distribution is consistent with prior reports in Santa Inês sheep [14], several breeds of calves [3, 24], Barbari goats [7], and adult cattle. These findings support the notion that hepatic topography is conserved among ruminants and reinforce the importance of this scanning window during abdominal ultrasonography. The extension of the liver to the sixth ICS places it in proximity to the lungs, which may result in misinterpretation in cases of pulmonary consolidation, especially by inexperienced operators, as consolidated lung tissue can mimic hepatic echotexture.

Subjectively, the liver exhibited homogeneous hypoechoic parenchyma, in agreement with findings in healthy ruminants and other species [7, 25, 26]. However, in pathological conditions such as hepatic lipidosis or chronic hepatitis, the liver parenchyma may become more heterogeneous and hyperechoic due to fat accumulation, fibrosis, and increased connective tissue [6].

Comparative analysis with the spleen showed that the liver was hypoechoic in most animals but occasionally isoechoic. This observation is clinically relevant, as conditions such as hepatic steatosis can increase hepatic echogenicity while leaving the splenic echotexture unchanged [27].

Measurements of the dorsal and ventral margins of the liver indicated that the organ was largest at the 10th and 11th ICS. Therefore, these ICS may be considered optimal sites for liver biopsy in goats due to the greater amount of accessible parenchymal tissue.

The CVC was observed with triangular morphology in the majority of animals, although rounded shapes were also present. This variability aligns with previous reports in calves and small ruminants [3, 7]. Although early studies suggested that loss of the triangular appearance was associated with hepatic congestion [1], recent research indicates that such variation can occur in healthy animals due to anatomical positioning in the vena caval groove [20].

The PV appeared as a circular, anechoic vessel with a hyperechoic wall and stellate branching, as observed in sheep [14]. The measurements of PV and CVC diameter and cross‐sectional area obtained in this study provide essential baseline values for assessing hemodynamic status and detecting conditions such as portal hypertension or right‐sided heart failure in goats.

Although both bile ducts and PVs appear as anechoic tubular structures in B‐mode ultrasonography, their differentiation in the present study was reliably achieved based on morphological criteria, particularly wall echogenicity, vessel diameter, and the characteristic stellate branching pattern of the portal venous system, which is absent in bile ducts.

The variability in gallbladder visualization underscores the need to interpret its absence cautiously, as it may not necessarily indicate pathology. The average gallbladder dimensions found in this study were greater than those reported in Barbari goats [7], likely reflecting differences in bile volume at the time of scanning.

In addition, gallbladder wall thickness proved to be a consistent ultrasonographic parameter, showing less variability than gallbladder size, which is influenced by physiological bile storage. Mild hypoechoic gallbladder content observed in a small proportion of animals was not associated with wall thickening or sedimentation and was considered a physiological variation in clinically healthy goats.

The findings of this study provide clinically useful reference values for the ultrasonographic evaluation of the liver, spleen, PV, CVC, and gallbladder in healthy lactating Saanen goats. These parameters can aid in the interpretation of organ size, echogenicity, and vascular architecture, especially in the early detection of hepatosplenic disorders.

Some limitations should be acknowledged. The study population consisted of animals from a single farm under intensive management, which may limit generalizability to goats under other production systems. Additionally, the absence of inter‐observer assessment restricts conclusions about repeatability. Finally, no pathological group was included, which limits direct comparisons between healthy and diseased states. Therefore, future research should explore these parameters in goats with confirmed hepatic or splenic disease to establish diagnostic cut‐off values and evaluate their dynamic changes in response to clinical interventions.

In conclusion, percutaneous ultrasonography of the liver and spleen in dairy goats is a highly feasible, safe, and noninvasive diagnostic technique. It can be readily performed on standing animals with minimal restraint, requiring only regional hair clipping and ultrasound gel for transducer‐skin contact. This method provides a reliable means of assessing organ size, structure, and parenchymal characteristics. The biometric reference values established in this study serve as important baseline data for clinical use and may support early detection of hepatosplenic abnormalities in small ruminants. Furthermore, these findings offer a foundation for future research on diagnostic protocols and disease monitoring, ultimately contributing to improved veterinary care and decision‐making in caprine medicine.

## Author Contributions

**Tatiane Vitor da Silva**: conceptualization, methodology, writing – original draft preparation, writing – review and editing. **Isabela Bernardes Moreira**: methodology and investigation. **Raissa da Silva Carvalho**: methodology and investigation. **Clara Beatriz de Avila Santiago**: methodology and investigation. **Mário Felipe Alvarez Balaro**: conceptualization, supervision, validation, writing – review and editing.

## Conflicts of Interest

The authors declare no conflicts of interest.

## Data Availability

The datasets generated during the current study are available from the corresponding author on request.
